# Host-Guest Interactions Between Metal–Organic Frameworks and Air-Sensitive Complexes at High Temperature

**DOI:** 10.3389/fchem.2021.706942

**Published:** 2021-08-03

**Authors:** Bo Huang, Zhe Tan

**Affiliations:** ^1^ Institute of Chemical Engineering and Technology, Xi’an Jiaotong University, Xi’an, China; ^2^ State Key Laboratory of Structural Chemistry, Fujian Institute of Research on the Structure of Matter, Chinese Academy of Sciences, Fuzhou, China

**Keywords:** HKUST-1, MIL-101, Fe(CO)_5_, chemical vapor infiltration, host-guest interaction, high temperature

## Abstract

The host-guest chemistry of metal–organic frameworks (MOFs) has been attracting increasing attention owing to the outstanding properties derived from MOFs-guests combinations. However, there are large difficulties involved in the syntheses of the host-guest MOF systems with air-sensitive metal complexes. In addition, the behaviors on host-guest interactions in the above systems at high temperature are not clear. This study reported the synthetic methods for host-guest systems of metal–organic framework and air-sensitive metal complexes via a developed chemical vapor infiltration process. With the synchrotron X-ray powder diffraction (XRPD) measurements and Fourier Transform infrared spectroscopy (FTIR), the successful loadings of Fe(CO)_5_ in HKUST-1 and NH_2_-MIL-101(Al) have been confirmed. At high temperatures, the structural and chemical componential changes were investigated in detail by XRPD and FTIR measurements. HKUST-1 was proven to have strong interaction with Fe(CO)_5_ and resulted in a heavy loading amount of 63.1 wt%, but too strong an interaction led to deformation of HKUST-1 sub-unit under heating conditions. NH_2_-MIL-101(Al), meanwhile, has a weaker interaction and is chemically inert to Fe(CO)_5_ at high temperatures.

## Introduction

Metal–organic frameworks (MOFs) or porous coordination polymers (PCPs) have attracted much attention in the past several decades (Zhang et al., 2020; Yuan et al., 2018). Benefiting from their high crystallinity, porosity, and designability, thousands of unique MOFs have been designed and synthesized, with potential applications in gas storage (Chen et al., 2020; Tian et al., 2018; Kalmutzki, Diercks, and Yaghi 2018; Alezi et al., 2015; Jiang et al., 2016), separation (Fu et al., 2016; Liu et al., 2018; Gao et al., 2020; Peng et al., 2017; Zhang et al., 2019), catalysis (Wang, An, and Lin 2019; Liang et al., 2018; Manna et al., 2015), and so on. On the other hand, metal complexes are one of the most developed research fields, with various properties such as magnetism (Guo, Bar, and Layfield 2019), superconductivity (Wang et al., 2019), and catalysis(Nicastri et al., 2020; Trammell, Rajabimoghadam, and Garcia-Bosch 2019). It would be useful to merge MOFs and metal complexes together and investigate their host-guest chemistry.

There have been several pioneering works in this field, but most research is focused on physical properties like electrical conductivity (Talin et al., 2014) and magnetism (Han et al., 2015). The interactions based on chemical properties between host MOFs and guest metal complexes are relatively less researched (Nayak, Harms, and Dehnen 2011). The largest limitation on research of host-guest systems for MOFs and metal complexes is the difficulties in their syntheses, especially in air-sensitive systems. In chemical processes, the chemical components and structures of them may have complicated changes. The systematic investigation on the host-guest chemistry of MOF-metal complex systems with air-sensitive complexes has rarely been reported, although a few air-stable metal complexes have already been researched, such as FeCp_2_ and Ru (cod) (cot) (Kalidindi, Yusenko, and Fischer 2011).

In this study, we reported a systematic research on MOF-metal complex systems of air-sensitive Fe(CO)_5_ guest in HKUST-1 and NH_2_-MIL-101(Al), consisting of syntheses, characterization, and investigation on host-guest interactions at high temperatures.

## Experimental

### Synthesis of HKUST-1

875 mg of Cu(NO_3_)_2_∙3H_2_O and 420 mg of benzene-1,3,5-tricarboxylic acid (BTC) were dissolved in 24 ml of H_2_O and EtOH mixture solvent (1:1 vol. ratio). The solution was sealed in a solvothermal container and reacted at 120°C for 12 h. The solution was then cooled down to room temperature at a fixed rate for 6 h. The light blue solid was filtered and washed with EtOH. The washed HKUST-1 was dried at 60°C and activated at 100°C under vacuum. The activated sample was stored in a glovebox for further use.

### Synthesis of NH_2_-MIL-101(Al)

Aluminium chloride hexahydrate (AlCl_3_∙6H_2_O, 0.51 g), 2-amino terephthalic acid (0.56 g), and DMF (30 ml) were heated at 130°C over 3 days. The product was refluxed in methanol overnight and dried at 100°C under vacuum overnight. The activated sample was stored in a glovebox for further use.

### X-Ray Powder Diffraction and Synchrotron X-Ray Powder Diffraction

The crystal structures of MOFs, Fe(CO)_5_-loaded MOFs, and their thermal decomposition products were investigated by powder XRD analysis using a Bruker D8 Advance diffractometer (Cu *Ka* radiation).

The crystal structures were investigated by capillary synchrotron XRPD analysis measured at the BL02B2 beamline, SPring-8. The XRPD patterns of the samples sealed in a glass capillary were measured *in situ* with a wavelength of 1.000 Å.

### Fourier Transform Infrared Spectroscopy and Transmission Electron Microscopy

FTIR spectra of MOFs, Fe(CO)_5_-loaded MOFs, and their thermal decomposition products were obtained to evaluate sample structures. All IR spectra were recorded inside a glovebox using a Bruker PLATINUM ATR FTIR spectrometer, accumulating 64 scans at a resolution of 4 cm^−1^.

TEM images were captured using Talos F200X operated at 200 kV accelerating voltage.

## Results and Discussion

### Chemical Vapor Infiltration of Fe(CO)_5_ Into HKUST-1

**FIGURE 1:**
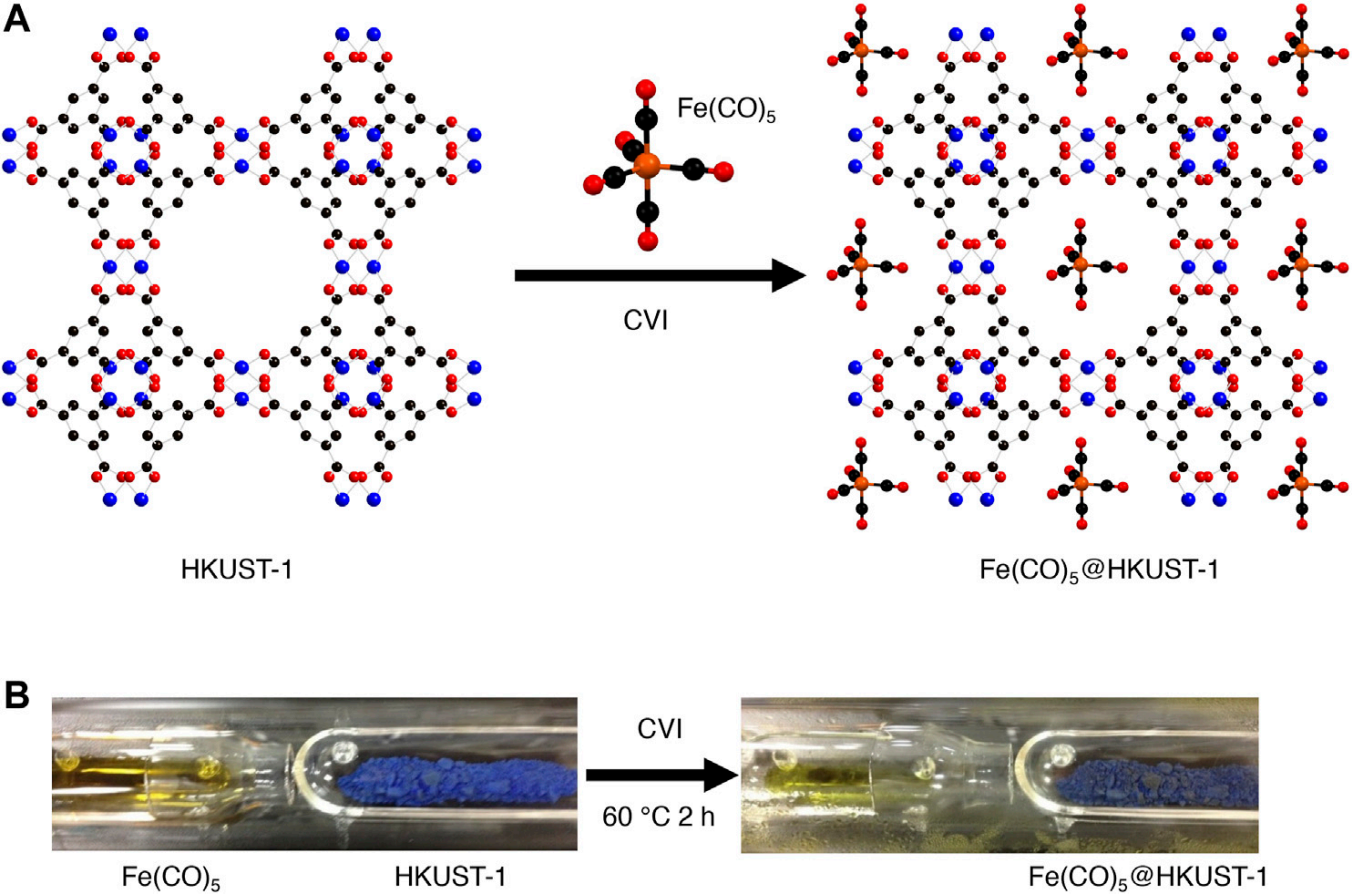
**(A)** Schematic image and **(B)** experimental images for Fe(CO)_5_ loading in HKUST-1 via a chemical vapor infiltration process.

Fe(CO)_5_ is one of the most common metal complexes which are sensitive to air. The Fe(CO)_5_ loading experiment in HKUST-1 was shown in the schematic image of Figure 1A via a chemical vapor infiltration (CVI) method. The HKUST-1 was synthesized and activated according to the reported method (Chui et al., 1999). The as-synthesized HKUST-1 was confirmed by capillary XRPD measurement; similar XRPD patterns with simulated HKUST-1 proved the successful synthesis of HKUST-1 (Supplementary Figure S1).

104.5 mg of activated HKUST-1 and 405.3 mg Fe(CO)_5_ were sealed in CVI vessel inside a glovebox (Figure 1B left). The CVI vessel was transferred to autoclave and heated at 60°C for 2 h for Fe(CO)_5_ loading; the obtained product was named Fe(CO)_5_@HKUST-1 (Figure 1B right). The mass of CVI product was weighed inside a glovebox as 270.3 mg, with 61.3 wt% loading amount.

**FIGURE 2:**
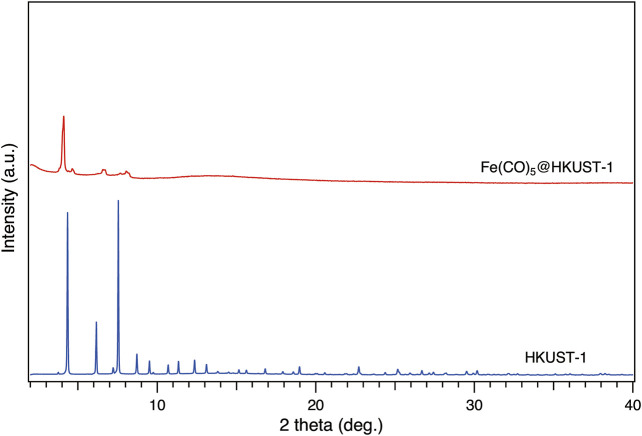
Synchrotron XRPD patterns of activated HKUST-1 (blue) and CVI product of Fe(CO)_5_@HKUST-1 (red) at 303 K. All samples were sealed in glass capillary in a glovebox under Ar atmosphere. The radiation wavelength was 1.000 Å.

To check the state of Fe(CO)_5_ loading in HKUST-1, synchrotron XRPD measurements have been carried out with incident wavelengths of 1.000 Å. All samples were kept under inert conditions inside glass capillary during measurements. As shown in Figure 2, the activated HKUST-1 exhibits strong diffraction intensity. However, after loading with Fe(CO)_5_, most of the diffraction peaks disappeared, with only remaining weak peaks below 10°. This phenomenon suggested the perturbation on the original crystalline structure of HKUST-1 by Fe(CO)_5_ infiltration.

To further confirm the MOF structure after loading, FTIR measurements have been performed under inert conditions. As shown in Supplementary Figure S2, sharp IR peaks were observed in activated HKUST-1. After loading of Fe(CO)_5_, all peaks originated from HKUST-1 remained, which strongly proved the stability of MOF structure before and after loading. In addition to those sharp peaks belonging to HKUST-1, a new typical carbonyl peak marked with asterisks has been found, confirming the successful loading of Fe(CO)_5_. Furthermore, the red shift of HKUST-1 peak at 1,650 cm^−1^ suggested the obstruction of MOF ligand vibration from the loaded Fe(CO)_5_ guest molecules, which gave further proof for successful loading of Fe(CO)_5_ inside HKUST-1 pores.

### High Temperature Behavior of Fe(CO)_5_@HKUST-1

In order to further understand the host-guest interactions between HKUST-1 and Fe(CO)_5_, we performed high temperature experiments with different heating conditions inside a glovebox. As shown in Supplementary Figure S3A, the dark blue solid of Fe(CO)_5_@HKUST-1 was heated at 140°C, and the color change to yellow was observed after 10 min heating (sample named **140–10**). We also tested other heating temperature of 170 and 200°C; similar color changes to yellow solid occurred within 1 min (Supplementary Figure S3B, samples named **170–1** and **200–1**, respectively). For longer heating times at 200°C, a gradual color change from yellow to purple was observed within 1.5 h (Supplementary Figure S3B, sample named **200–90**).

**FIGURE 3:**
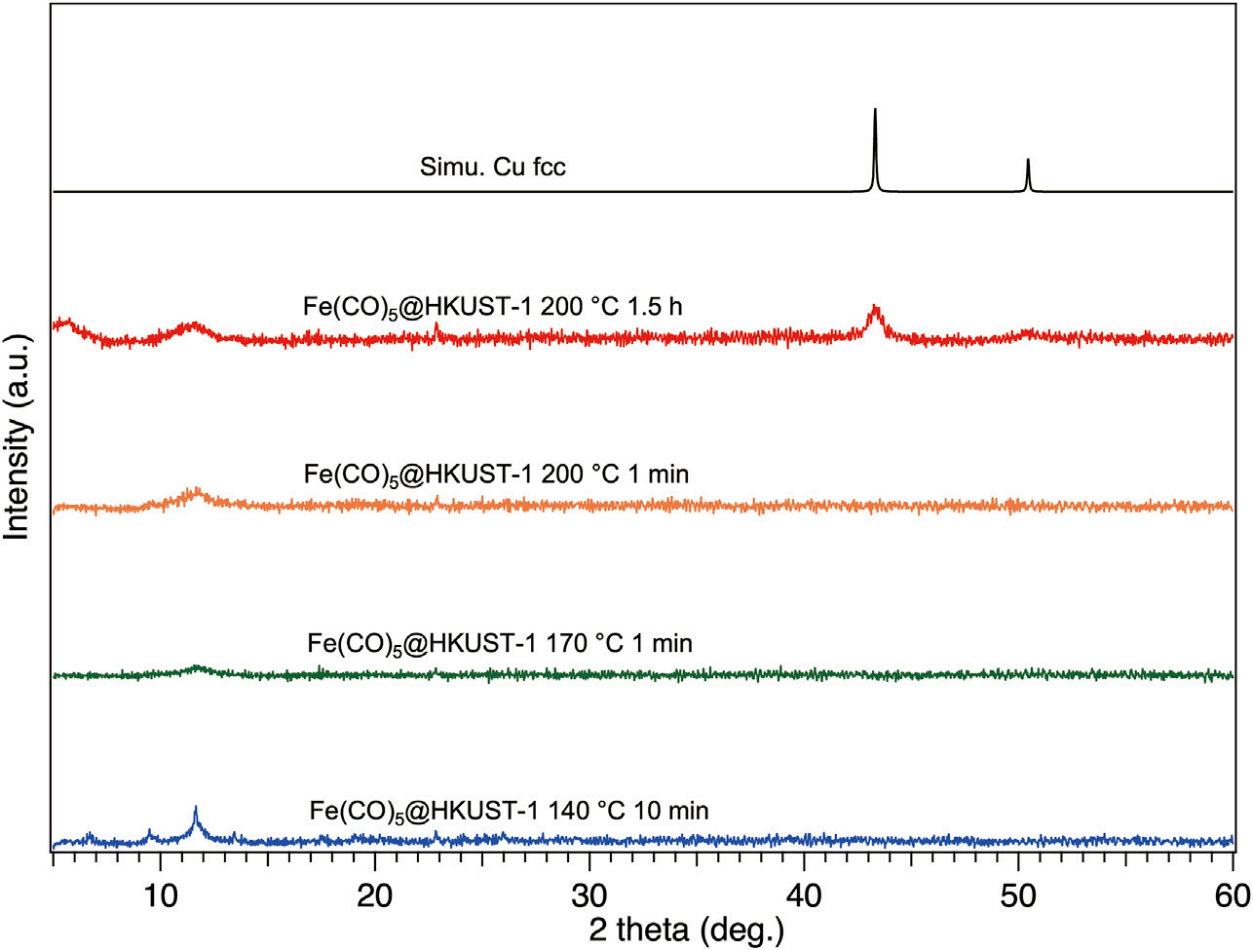
XRPD patterns of Fe(CO)_5_@HKUST-1 decomposed at different conditions: 140°C-10 min (blue), 170°C-1 min (green), 200°C-1 min (orange), 200°C-1.5 h (red), and simulated Cu (black). All samples were sealed inside a glovebox and measured under inert conditions. The background of XRPD patterns had been removed for clarity. The radiation wavelength was 1.5406 Å.

To explore the structural information of Fe(CO)_5_@HKUST-1 under various heating conditions, we performed XRPD measurements under inert conditions by using an inert sample holder (Supplementary Figure S4). Before measurements, to avoid the samples being contaminated by air, the inert holder was tightly sealed inside a glovebox after sample setup. The measurement results are shown in Figure 3; after heat treatment at 140°C for 10 min, the crystallinity of HKUST-1 was slightly damaged compared with XRPD pattern of Fe(CO)_5_@HKUST-1 in Figure 2, due to the thermal decomposition of Fe(CO)_5_. In TEM image of **140–10**, a homogenous solid structure was observed (Supplementary Figure S5), the result is consistent with its XRPD pattern. For **170–1**, even the heating time was much shorter than **140–10**, and higher temperatures led to harsh damage on crystallinity, where only the (222) main peak at 11.8° of HKUST-1 remained, shown in Figure 3, owing to faster decomposition of Fe(CO)_5_. The XRPD result of **200–1** was similar to that of **170–1** (Figure 3).

With the extension of heating time to 1.5 h (sample **200–90**), a crush of crystallinity was accompanied with the appearance of new broad diffraction peaks at 43.5 and 50.7°, which are consistent with diffraction peaks of simulated face-centered cubic (fcc) Cu, proving the generation of Cu NPs. Actually, under vacuum conditions, after heating at 200°C for 18 h, no Cu diffraction peak was detected (Supplementary Figure S6). Even with sufficient heating of HKUST-1 at 500°C for 2 h (Lee et al., 2019), instead of the metallic state of Cu NPs, only semiconductor CuO NPs can be obtained. On the other hand, Cu NPs can be easily generated when the heating atmosphere is changed to 1 atm of H_2_, at 200°C for 1 h (Supplementary Figure S7). Under H_2_ atmosphere, the decreasing on (222) peak of Cu^2+^ ion-rich plane in HKUST-1 and the increasing on (111) peak of Cu NPs demonstrate the migration of Cu elements from the square-planar coordinated Cu^2+^ ions in HKUST-1 to dodecahedral coordinated Cu atoms in fcc-Cu NPs. Therefore, in sample **200–90**, it may be suggested that the reduction reaction of Cu^2+^ to Cu atom was triggered by decomposition of Fe(CO)_5_. The existence of Cu NPs in **200–90** was also confirmed by TEM measurement (Supplementary Figure S8).

**FIGURE 4:**
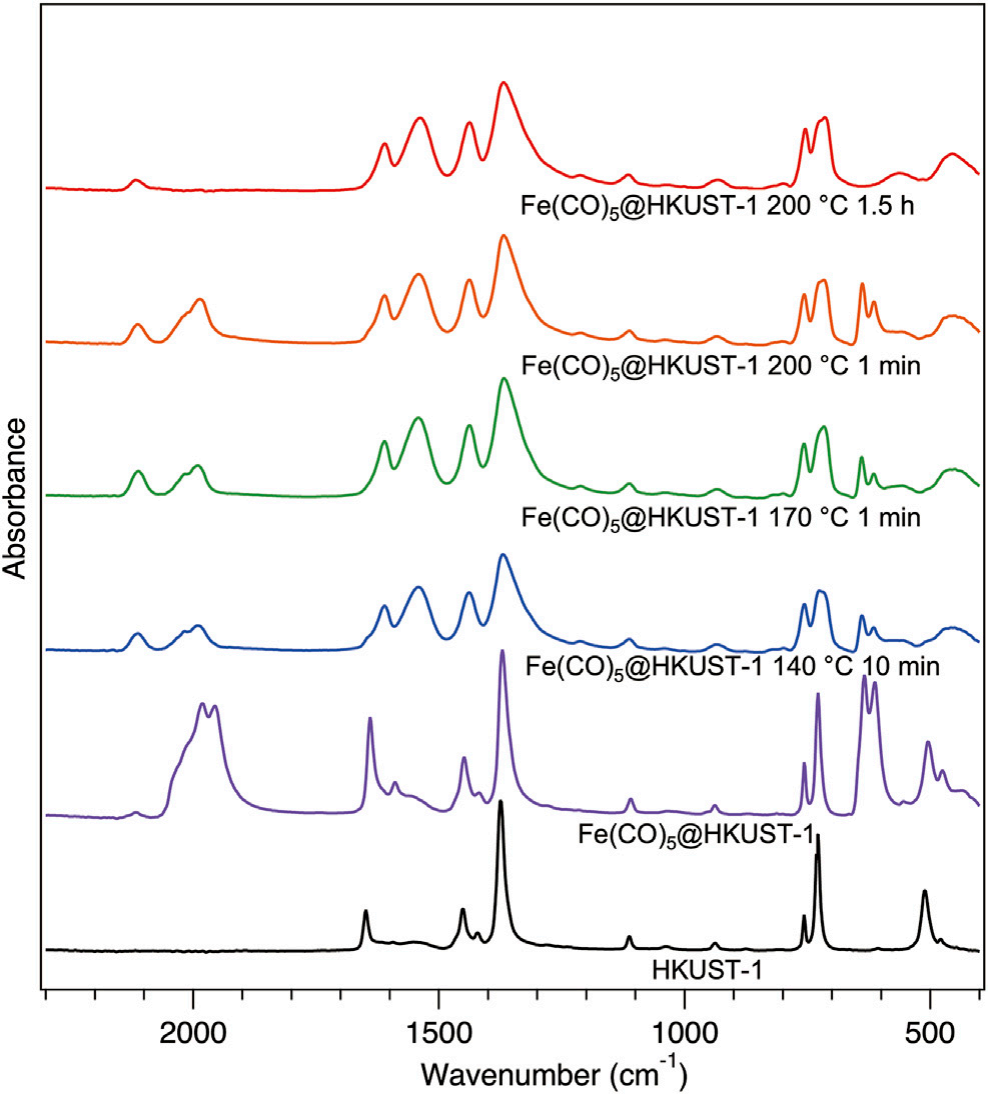
Infrared spectra for activated HKUST-1 (black), Fe(CO)_5_@HKUST-1 (purple), and the thermal decomposition products of Fe(CO)_5_@HKUST-1 at different conditions: 140°C-10 min (blue), 170°C-1 min (green), 200°C-1 min (orange), and 200°C-1.5 h (red). All samples were measured inside a glovebox.

To understand more about the thermal decomposition processes of the Fe(CO)_5_@HKUST-1 at various conditions, the FTIR spectroscopy has been measured inside a glovebox (Figure 4). Samples **140–10**, **170–1,** and **200–1** show almost identical IR spectra among each other. The incomplete decomposition of Fe(CO)_5_ can be noticed from the remaining typical CO peaks in these three samples. The broadening of MOF peaks compared with the initial sharp peaks from HKUST-1 demonstrated the decomposition of MOFs sub-units under thermal decomposition of Fe(CO)_5_ guest molecule, in addition to the collapse of HKUST-1 crystal structure confirmed by XRPD measurements in Figure 3B. In spectrum of **200–90**, CO peaks vanished because of the complete decomposition of Fe(CO)_5_ with a longer heat treatment.

### High Temperature Behavior of Fe(CO)_5_@MIL-101(Al)

The above results have shown the host-guest interactions in case of Fe(CO)_5_@HKUST-1 at high temperatures, where the decomposition of Fe(CO)_5_ broke the Cu_2_O_8_ clusters in HKUST-1. Other MOFs with different clusters may resist Fe(CO)_5_ at high temperatures. Among potential candidate MOFs, we guessed that NH_2_-MIL-101(Al) can be durable to Fe(CO)_5_ at high temperaturse because of its relatively high thermal stability and chemical inertness. The NH_2_-MIL-101(Al) was synthesized via a reported protocol (Serra-Crespo et al., 2011).

After activation of NH_2_-MIL-101(Al), the gas phase loading of Fe(CO)_5_ to NH_2_-MIL-101(Al) was performed by CVI process under similar conditions. 101.7 mg of activated NH_2_-MIL-101(Al) and 403.2 mg Fe(CO)_5_ were kept at 60°C for 2 h under Ar atmosphere. The mass of loading product Fe(CO)_5_@MIL-101(Al) was 127.4 mg with 20.2 wt% loading amount. Lower loading amounts for NH_2_-MIL-101(Al) than 61.3 wt% of HKUST-1 may be attributed to its weaker host-guest interaction as the lack of open metal site.

**FIGURE 5:**
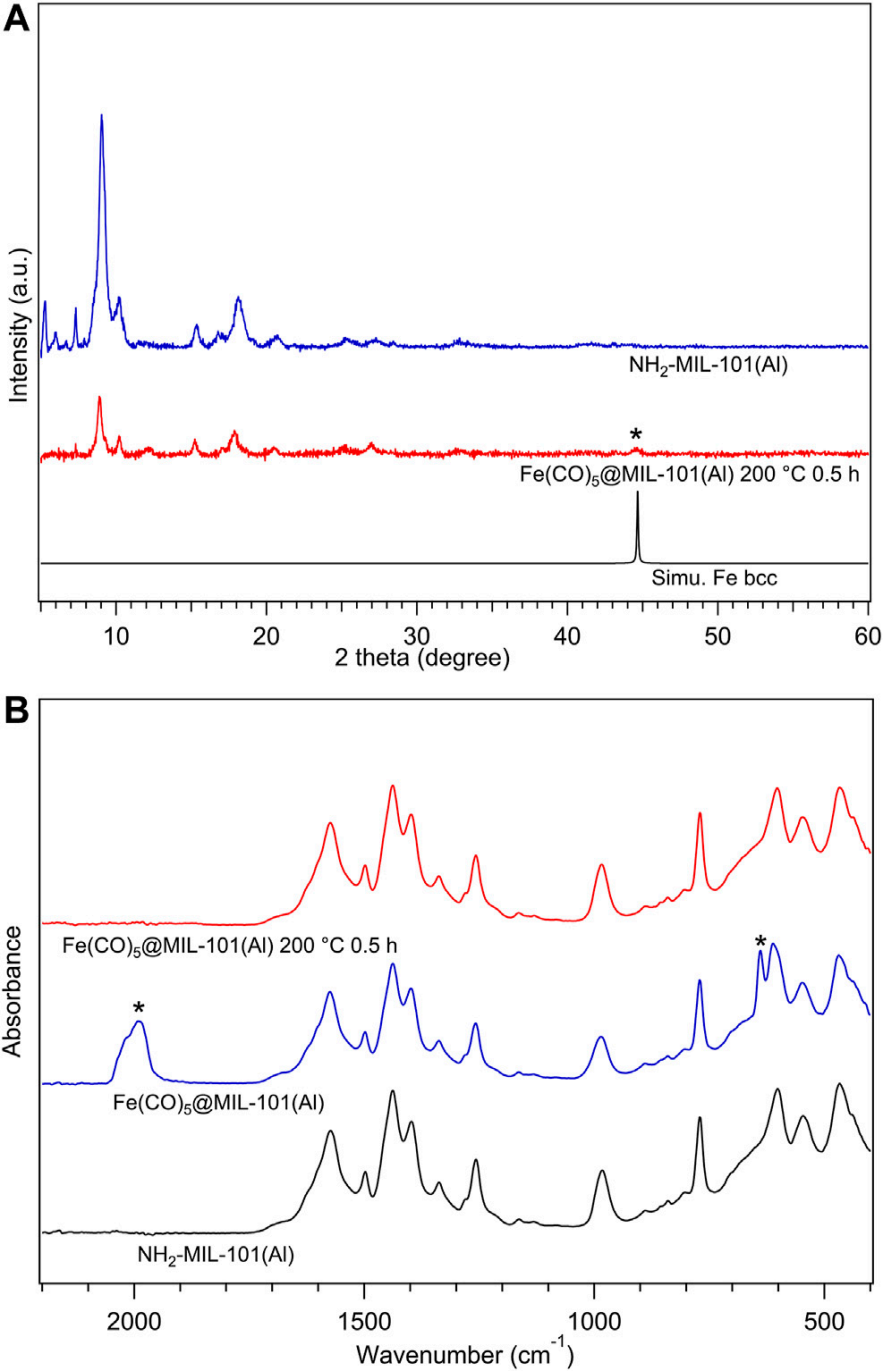
**(A)** Infrared spectra for activated NH_2_-MIL-101(Al) (black), Fe(CO)_5_@MIL-101(Al) (blue), and Fe(CO)_5_@MIL-101(Al) decomposed at 200°C/0.5 h (red), all samples were measured inside a glovebox. **(B)** XRPD patterns of NH_2_-MIL-101(Al) (blue), Fe(CO)_5_@MIL-101(Al) decomposed at 200°C/0.5 h (red), and simulated body-centred-cubic Fe (black), all samples were measured under inert conditions by capillary technique. The radiation wavelength was 1.5406 Å.

The CVI product Fe(CO)_5_@MIL-101(Al) was investigated by FTIR measurements performed inside the glovebox. As shown in Figure 5A, there are typical carbonyl vibration peaks in the spectrum of Fe(CO)_5_@MIL-101(Al) marked with asterisks, proving the existence of Fe(CO)_5_ guest molecules in the product. Combined with the carbonyl peaks, the sustenance for the IR peaks of the MOF structure before and after CVI process suggested the successful loading of Fe(CO)_5_ to NH_2_-MIL-101(Al) without breaking the MOF structure.

The thermal stability of Fe(CO)_5_@MIL-101(Al) was tested under inert conditions. Fe(CO)_5_@MIL-101(Al) was heated to 200°C (same temperature as Fe(CO)_5_@HKUST-1) for 0.5 h, referred to as **200–30**. In Figure 5A, with the comparison of the FTIR spectra of NH_2_-MIL-101(Al), Fe(CO)_5_@MIL-101(Al), and **200–30**, the complete decomposition of Fe(CO)_5_ and the maintaining of MOF structure can be confirmed in **200–30**. On the other hand, in XRPD measurement results shown in Figure 5B, the asterisk diffraction peak from the body-centred cubic (bcc) Fe NPs was detected in **200–30**, as solid evidence for Fe formation from Fe(CO)_5_. The XRPD measurements also show the lower crystallinity in diffraction pattern of **200–30** compared with that of NH_2_-MIL-101(Al), which may be considered as a perturbation of periodic structure by randomly dispersed guest molecules inside pores.

Finally, to elucidate the reasons for the different behaviors between Fe(CO)_5_@HKUST-1 and Fe(CO)_5_@MIL-101(Al), the possible reaction mechanisms in each case should be further discussed. Since the decomposition of Fe(CO)_5_ below 150°C is negligible (Carlton and Oxley, 1965), the molecular Fe(CO)_5_ reacting with HKUST-1 backbone in **140–10** can be considered as the redox reaction between penta-coordinated Fe (0) with tetra-coordinated Cu(II) open metal sites. At higher temperatures of 170 and 200°C, the Fe(CO)_5_ decompositions were significantly promoted, supplying atomic Fe and molecular CO to these systems. HKUST-1 backbone is stable with CO molecule to at least 210°C, (Stawowy et al., 2020), therefore, the rapid destruction of MOFs shown in Figure 4 for **170–1** and **200–1** are mainly attributed to the strong reduction ability of atomic Fe. As the ligand reactivity difference between benzene-1,3,5-tricarboxylic acid in HKUST-1 and 2-amino terephthalic acid in NH_2_-MIL-101(Al) is almost negligible, the main reason for the different behavior of Fe(CO)_5_@HKUST-1 and Fe(CO)_5_@MIL-101(Al) can be concluded as the different reactivity of metal ions. With the systematic investigations on loading and heating processes of Fe(CO)_5_ guest to HKUST-1 and NH_2_-MIL-101(Al), the interactions between host MOFs and guest molecules were clarified.

## Conclusion

The host-guest systems of MOFs and air-sensitive metal complexes were synthesized, with Fe(CO)_5_ loaded in HKUST-1 and NH_2_-MIL-101(Al) via a chemical vapor infiltration process. With the XRPD and FTIR measurements under inert conditions, the successful loading of Fe(CO)_5_ in HKUST-1 and NH_2_-MIL-101(Al) has been confirmed. The interactions between MOFs and Fe(CO)_5_ at different heating temperatures were investigated in detail. As a result, the Fe(CO)_5_@HKUST-1 was not stable under thermal conditions. Under minor heating conditions, deconstructions of HKUST-1 sub-unit were observed from XRPD and FTIR results; under major heating conditions, Cu^2+^ ions in HKUST-1 were reduced to Cu NPs with the interaction to Fe(CO)_5_. On the other hand, in Fe(CO)_5_@MIL-101(Al), the decomposition of Fe(CO)_5_ to Fe NPs was found and NH_2_-MIL-101 (Al) was stable to Fe(CO)_5_ as well as its decomposition products. The synthetic methods and the systematic investigation for the air-sensitive host-guest MOFs-metal complex systems in this report provide valuable experimental experience and insight between porous functional materials and guest molecules. We envision that this method could be expanded to other metal precursors and porous materials on fabricating various functional nanocomposites and devices.

## Data Availability

The original contributions presented in the study are included in the article/Supplementary Material, further inquiries can be directed to the corresponding author.
