# Supplementary material for: Host-Guest Interactions Between Metal–Organic Frameworks and Air-Sensitive Complexes at High Temperature
Source: Front Chem. 2021 Aug 3;9:706942. doi: 10.3389/fchem.2021.706942 (PMC8369409; doi:10.3389/fchem.2021.706942)
Supplement: Supplementary file 1 [file DataSheet1.PDF]

Supplementary Material

Host-Guest Interactions between Metal-Organic Frameworks and Air-Sensitive Complex at High Temperature

Bo Huang*, Zhe Tan

*Correspondence and requests for materials should be addressed to

B. Huang (email: bohuang@xjtu.edu.cn).

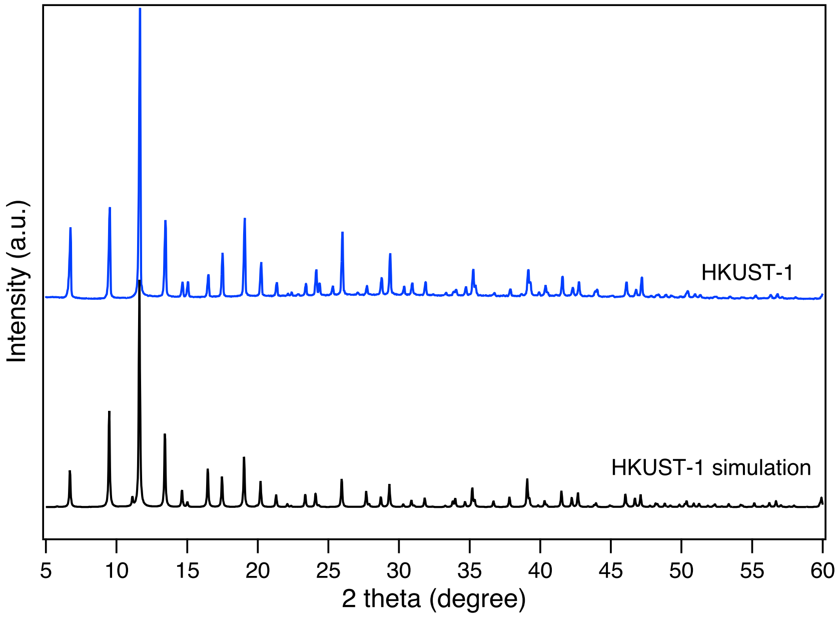

Supplementary Figure 1. XRPD patterns of as-synthesized and simulated HKUST-1. The radiation wavelength was 1.5406 Å.

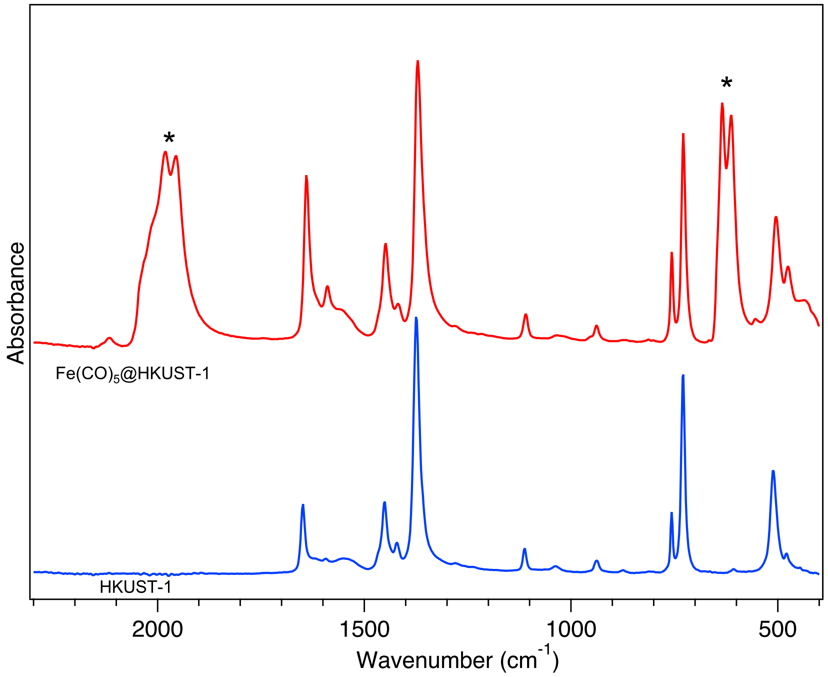

Supplementary Figure 2. FTIR spectra of activated HKUST-1 and $Fe(CO)_5$@HKUST-1. The asterisks show additional peaks from $Fe(CO)_5$.

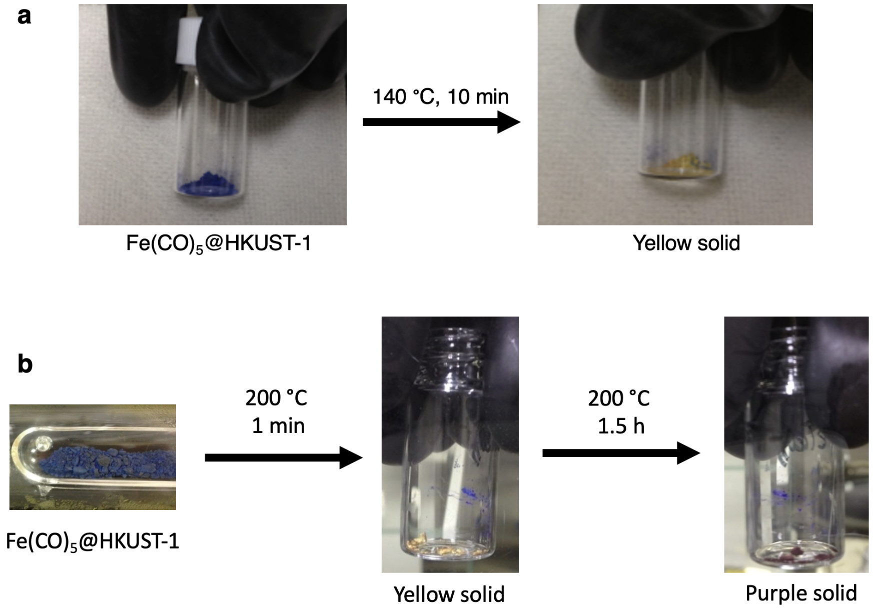

Supplementary Figure 3. Thermal stability experiments of $Fe(CO)_5$@ HKUST-1 under inert condition at (a) 140 ºC for 1 min; (b) 200 ºC for 1 min and 1.5 h.

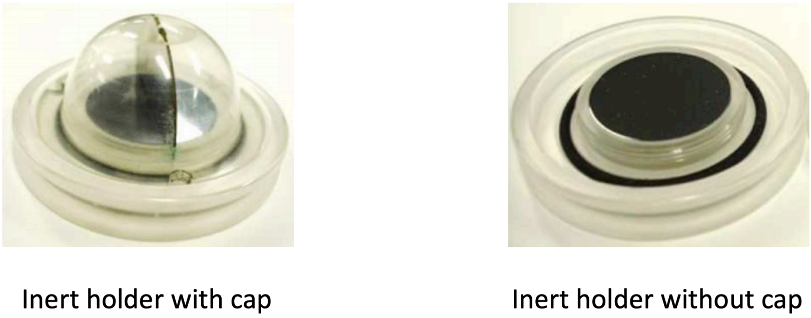

Supplementary Figure 4. The images of inert holder used in XRPD measurements.

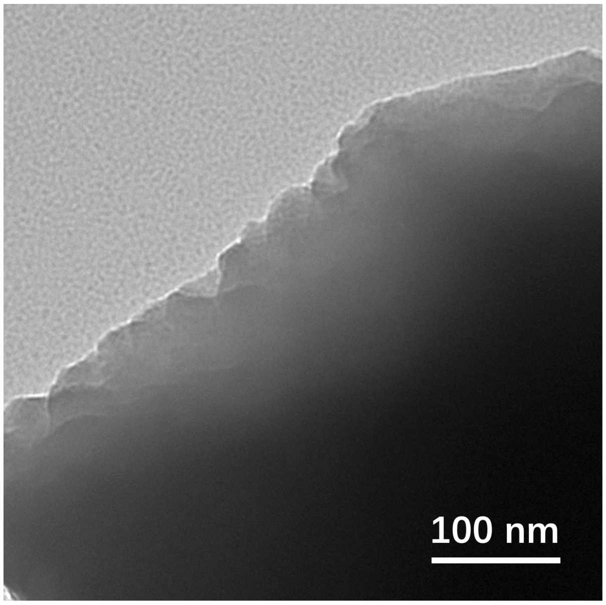

Supplementary Figure 5. The TEM image of $Fe(CO)_5$@ HKUST-1 heated at 140 ˚C for 10 min.

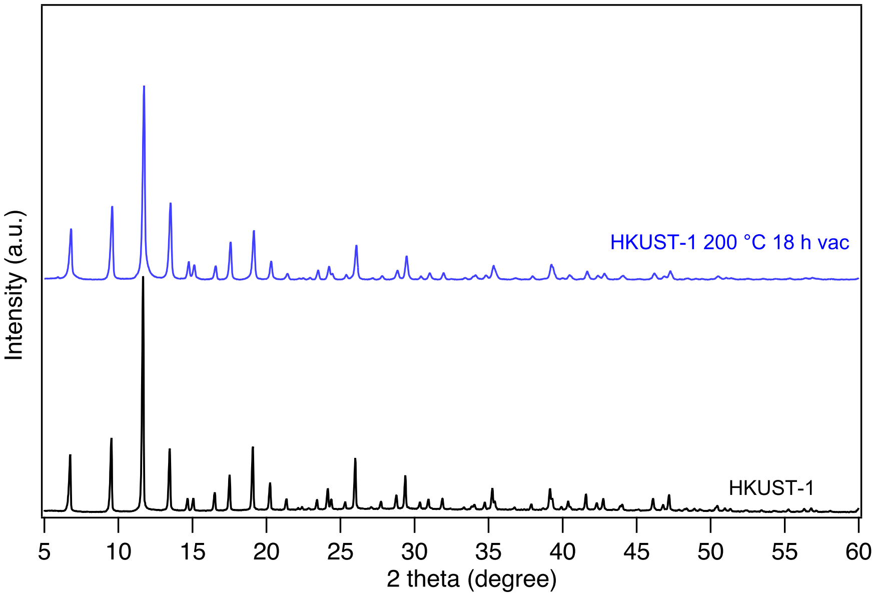

Supplementary Figure 6. XRPD patterns of HKUST-1 and HKUST-1 heated at 200 ˚C for 18 h under vacuum. The radiation wavelength was 1.5406 Å.

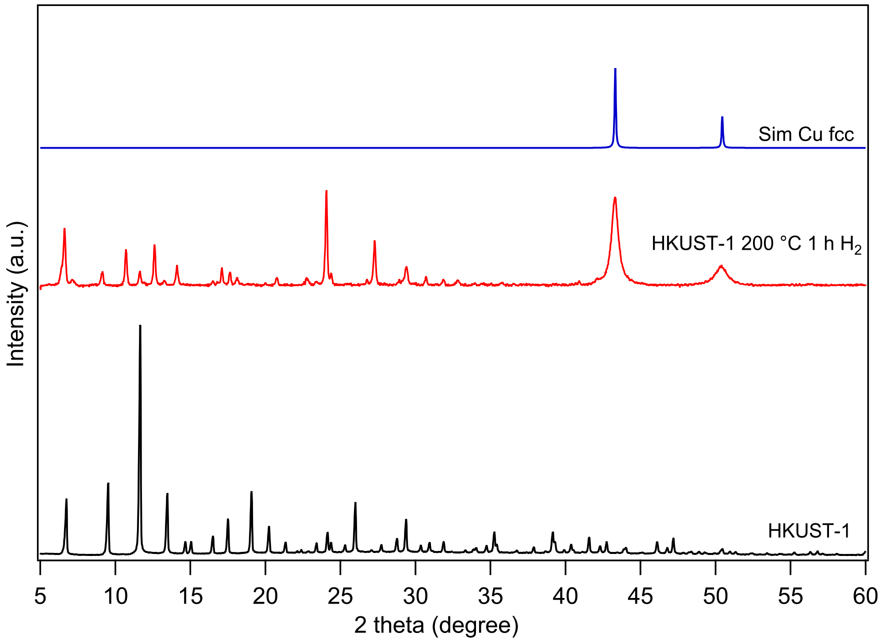

Supplementary Figure 7. XRPD patterns of HKUST-1, simulated fcc-Cu and HKUST-1 heated at 200 ˚C for 1 h under 1 atm H_2 atmosphere. The radiation wavelength was 1.5406 Å.

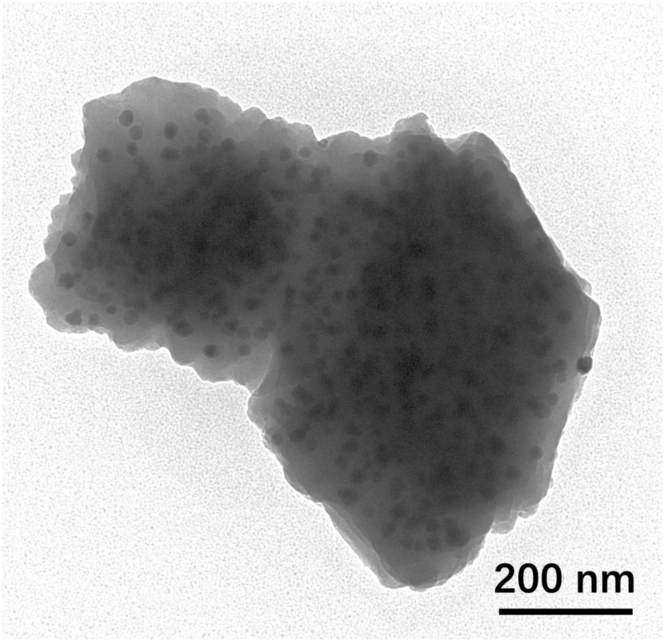

Supplementary Figure 8. The TEM image of $Fe(CO)_5$@HKUST-1 heated at 200 ˚C for 1.5 h.
